# Metabolomic Studies for Metabolic Alterations Induced by Non-Steroidal Anti-Inflammatory Drugs: Mini Review

**DOI:** 10.3390/biom11101456

**Published:** 2021-10-04

**Authors:** Soumita Ghosh

**Affiliations:** Department of Systems Pharmacology and Translational Therapeutics, Perelman School of Medicine, Institute for Translational Medicine and Therapeutics, University of Pennsylvania, Philadelphia, PA 19104, USA; soumita@mail.med.upenn.edu

**Keywords:** metabolomics, non-steroidal anti-inflammatory drugs, pathway analysis

## Abstract

Non-steroidal anti-inflammatory drugs (NSAIDs) are Food and Drug Administration (FDA) approved antipyretic, anti-inflammatory, and analgesic drugs to mitigate pain, however it is associated with gastrointestinal injury and cardiovascular disease in some individuals. Metabolomics has the potential to understand the interaction of host and the drugs, such as NSAIDs administration. This discipline has been used by many researchers to understand the serious side effects of NSAIDs. We highlighted (1) the potential of metabolomics in understanding the pathogenesis of adverse events due to NSAIDs administration; (2) choice of metabolomics techniques, bio sample handling; (3) review of metabolomics studies in the front of NSAIDs in different biofluids and tissues; (4) pathway analysis of the data presented in the published literature. In our analysis we find tricarboxylic acid cycle (TCA), “glycine serine and threonine metabolism,” “alanine, aspartate, and glutamate metabolism,” and fatty acid metabolism to be altered by the NSAIDs like ibuprofen, indomethacin, naproxen, aspirin, and celecoxib. In conclusion, metabolomics allows the use of biological samples to identify useful pathways involved in disease progression, and subsequently inform a greater understanding of the disease pathogenesis. A further in-depth investigation of the associated pathways mentioned above holds the potential for drug targets for side effects mitigation.

## 1. Introduction

Metabolomics is a top-down system biological tool to study metabolome. This field consists of the application of statistical tools on the data obtained by advanced analytical platforms on bio-fluids or tissues. The metabolome is the collection of endogenous small molecules like amino acids, fatty acids, breakdown products of amino acids, etc., of cellular biochemistry or that derived from host microbiota under normal or abnormal cellular processes [1]. These molecules are products of chemical reaction in a biological system. Further these molecules have widespread functions in the host like signaling, development, and physiological functions. It is thus a comprehensive study of the overall metabolism [2]. Due to its proximity to phenotype, metabolome is often used for the biomarker-determination of a disease.

NSAIDs constitute for 5% of all the drugs sold over the counter. Despite being popular drugs for the mitigation of pain and inflammation, these drugs are, however, known to cause several side effects like gastrointestinal (GI) pathology [3,4] and cardiovascular disease [5]. 40% of all the NSAIDs users have symptoms like gastroesophageal reflux and dyspeptic symptoms [6]. The onset of the symptoms depends on the nature of the NSAIDs consumed. Studies showed that Cyclooxygenase-2 (COX-2)-selective NSAIDs were associated with less symptomatic and endoscopically detected ulcers as opposed to non-selective NSAIDs like naproxen, ibuprofen etc. The symptoms were not predictive of the presence of mucosal injury. While 50% patients who exhibited symptoms had no mucosal injury, more than 50% had peptic ulcers and had no previous symptoms [7].

Small intestine inflammation is present in 60–70% of long-term NSAIDs users while perforations in 50–70% [8]. Furthermore, although COX-2 selective drugs reduce the incidence of GI effects, they are associated with adverse cardiovascular events [3]. Previous reports demonstrated the role of NSAIDs toward blood pressure elevation, heart failure, sudden cardiac death [5]. 40% NSAIDs users are at risk of receiving diagnosis of hypertension [9].

Some groups have demonstrated the role of arachidonic acid (AA) derivatives for the adverse cardiac functions [10,11]. Often, the drug’s adverse effects like gut damages are asymptomatic and are associated with life-threatening ulceration and bleeding before they could be detected. Thus, there is an urgent need to personalize the drugs according to specific needs and susceptibility of the patients toward the side effects. Consequently, early detection of GI damage or cardiovascular disease through biomarker discovery and related pathophysiological mechanisms are critical in controlling the associated complications. Metabolic fingerprints in biofluids and tissues would help to inform practical applications as a next-generation tool for offering solutions to problems in NSAID-related side effects. This review summarizes the progress of metabolomics, in murine response to NSAIDs administration. We also discuss the associated underlying mechanisms and related pathways, the potential clinical challenges, and future perspectives and suggestions for the further research endeavors in this front.

## 2. Choice of Metabolomics Techniques, Study Design and Bio-Sample Handling to Conduct Metabolomics Study to Understand Metabolic Alterations by NSAIDs

### 2.1. Metabolomics and Metabolic Fingerprinting Techniques

Metabolome is very sensitive to internal and external stressors and thus is a probe to phenotype. The metabolic readout of the host is referred to as “metabotype” [12]. Metabolomics combined with other “omics” like proteomics; genomics can potentially elucidate the pathways involved in the process in a comprehensive way.

The detection and analyses of metabolites is complex. Various analytical platforms are used in this field [13]. There has been an immense development of the technologies used for metabolomics which has consequently led to the development of detection of the less abundant molecules reliably in biofluid mixture. Nuclear magnetic resonance (NMR), liquid chromatography (LC), gas chromatography (GC) coupled to mass spectrometry (MS) and capillary electrophoresis (CE) have been used in metabolomics for the purpose of metabolite quantification [14] as well as structural characterization [15]. Each technique has its own advantages and disadvantages. NMR is widely used in metabolomics for reproducibility, ease in sample handling and non-destructive nature of the sample assessment. Further, only one internal standard is enough for the quantitation of all the molecules present in the sample which makes this technique cost effective. NMR in metabolomic experiments rely on ^1^H NMR spectroscopy, as all the metabolites hold ^1^H nucleus. For the metabolomics study, most extensively used pulse sequence is the first transient of the NOESY (Nuclear Overhauser Effect spectroscopy). The pulse sequence is (RD-90°-τ-90°- τmix-90°-ACQ), where RD = interscan relaxation delay, τ = short delay, and τmix = mixing time. This pulse sequence is abundantly used for urine and in tissue extracts free of proteins. This pulse program provides good water suppression and produce reproducible results [16].

In serum and plasma, presence of proteins and other large molecules causes a significant challenge to observe the metabolite signals. In such cases, specific pulse sequences such as CPMG (Curr-Purcell-Meiboom-Gill) are used, that attenuate the broad signals from the macromolecules [17]. The detection of metabolite signal by higher dimensionality NMR like 2D COSY, TOCSY has significantly improved the quantitation of overlapped signal in the spectra [18]. Contrary to NMR, mass spectrometry (MS) technique is used to detect low abundant molecules in the system [19,20]. It is popular for its high sensitivity and high throughput nature. However, since it uses ionization technique, the sample is often lost in the analysis. Nevertheless, since very small volume of samples are injected, the loss is often irrelevant to the user. The MS is often coupled with (LC), (GC), or (CE). LC requires the use of columns to separate the compounds based on the polarities. The most used ionization in LC is electrospray ionization (ESI). ESI being soft ionization technique facilitates the determination of molecular mass of parent ion. While Triple Quad and Ion trap are often used as mass analyzers for the targeted metabolomics, TOF analyzers are used for untargeted metabolomics. Following the spectra acquisition, the analysis follows a pipeline. NMR spectra is generated with signature from several compounds. Thus, a database containing the spectral profiles of pure compounds is matched to the mixtures of the biofluid to get specific information [21,22]. Further, untargeted metabolomics involves the workflow with global profiling, identification of peaks in spectra, and quantitation of the peaks. In targeted profiling, the area under the peak is normalized to peak area of the internal standard under consideration for quantitation. Following peak quantitation in all platforms, the data are analyzed using different multivariate tools like principal component analysis (PCA) and partial least square discriminant analysis (PLS-DA) [23].

Metabolomics addressed various biological questions over the decades, like studying global effects of genetic manipulations [24], diseases [25,26,27], disease prediction [28]; the review discusses all the studies that have used metabolomics to explore the possible metabolic effects due to administration of nonsteroidal anti-inflammatory drugs (NSAIDs). The review primarily discusses the studies to enhance our understanding on the metabolic effects of NSAIDs by analyzing the pathways involved in various studies. The data in each paper are used to perform “pathway analysis” which is further studied in detail to see if common pathways are affected in each of those studies.

### 2.2. Study Design of the Metabolomics Experiment in NSAIDs Based Toxicology Study

Like other clinical and pre-clinical investigations, metabolomics studies should have a well-defined question to address. The design must clearly include subjects or model organisms that have received certain drugs and that there should exist a placebo/control group. A well-planned study that aims to examine NSAIDs response should have multiple doses and multiple time points for sample collection in humans. However, acute GI injury can be invoked by a single dose of NSAIDs like indomethacin, ibuprofenprofen, and naproxen at the doses of 25 mg/kg, 800 mg/kg, and 100 mg/kg respectively when administered orally to rats. It is relevant to explore the metabolic profile in biofluids and tissue samples at different time points post drug administration as that would shed light on the metabolic response in context of drug concentration in host. Cardiovascular effects are induced by chronic administration of COX-2 selective inhibitors like rofecoxib at 50 mg/L for 3 months in mice. Moreover, including different types of NSIADs will further incorporate the specificity of the result toward a certain type of NSAIDs. In order to explore biomarkers specific to gut damage, biofluids such as urine or serum are required to be collected for exploration. However, to understand the mechanistic aspect of the GI lesion or cardiovascular disease, specific tissues should be collected for metabolite profiling which will provide understanding of the disease pathophysiology.

Exposure to NSAIDs causes a change in the gene expressions [29], protein expression [30], and further metabolite level change in the host [31]. This discipline focusses on understanding the effects of NSAIDs on small molecule component of biofluids. Most important and relevant question in the field is to understand the molecular signatures due the metabolic perturbations by NSAIDs which will further lead to biomarker discovery and related mechanistic details.

### 2.3. Sample Preparations and Storage for Metabolomics Experiment

For metabolomics studies, appropriate sample storage procedures should ensure that all samples are treated in the same manner. The first step in this field is to identify the biofluid that would answer the specific biological question at hand. Sample handling is very important in this field as metabolites are affected by mishandling of the samples and poor storage conditions [32]. It is thus crucial to snap freeze the samples immediately after dissection to minimize factors such as, freeze-thaw cycles and contamination and to reduce small changes to the metabolic profiles. Freezing immediately is important as some metabolites degrade very rapidly if enzymatic activity is not stopped completely. Thus, the tissues should be frozen at –80 °C till the extraction as that preserves the integrity of the metabolome [33]. Sample preparation is also a time-consuming as well as error-prone bio-analytical step, particularly when handling complex biological matrices such as blood fluids [34]. Consequently, no universal technique suitable for blood fluid sample preparation for metabolomic fingerprinting exists [35]. Urine should be collected in azide solution to minimize bacterial growth [36]. Any bacterial growth in urine can potentially change the molecules of interest. Biological samples for metabolomics analyses require a standardized extraction protocol in laboratory settings with associated techniques available. For targeted assay, the protocol should be validated for measurement of metabolites under considerations. Pooled quality control, blanks must be included with each run to monitor the variability in extractions, acquisitions etc. Depending on tissue/biofluid under consideration, and the biological question asked, the analytical platform should be fixed, and the samples must be processed.

## 3. Metabolomics of Murine Response toward NSAIDs Administration

Although NSAIDs are used worldwide as anti-inflammatory drugs, it is not without side effects. One of the failures in this front of eradicating the side effects of the drugs is due to the lack of proper understanding of drug–host interaction. While it is challenging to study the side effects of drugs in humans, murine subjects provide a suitable alternative. This is because murine subjects belong to homogenous genetic background, and they thrive in controlled conditions in laboratory. These models offer the perfect opportunity to follow the metabolic effects in different treatments with parallel monitoring of their histological changes. Drug–host interaction is very similar in humans as in rats like GI lesions and myocardial infraction. Theoretically, the biomarkers obtained in the rodents should have a translational value, however before using in clinical settings the biomarkers should be validated in humans.

Nine studies (2011–2021) have been performed in the front of metabolomics and NSAIDs. Three of the studies used targeted lipidomics approach [10,37,38] while rest six studies used the approach of untargeted metabolomics [39,40,41,42,43,44]. All studies involved murine subjects. Most studies are centered around the biofluid explorations like urine and serum for biomarker discovery. Further, two studies used stomach tissue to understand the molecular level perturbations in stomach tissues by NSAIDs administration. Overall, the studies show metabolic reprogramming due to NSAIDs administration and there are common pathways affected in different studies.

### 3.1. Lipidomics Signature in NSAIDs Administration

Three studies used targeted lipidomics approach [10,37,38]. Ref. [10] applied lipidomic profiling in mouse model. The researchers measured 27 oxylipins and identified lipidomic fingerprint in rofecoxib and detrimental cardiovascular events. They found a significant increase in 20-hydroxyeicosatetraenoic acid (20-HETE) in plasma, a causal molecule for risk of myocardial infraction (MI) and stroke. The investigators reported an increase of 120-fold for 20 HETE in rofecoxib-administered mice. Additionally, administration of 20-HETE reduced the clotting time for blood. 20-HETE was therefore hypothesized to be the biomarker for cardiovascular disease. In a separate study, Li et al. [37] demonstrated the beneficial effects of elevated epoxyeicosatrienoic acid (EETs) levels and the EETs/dihydroxyeicosatrienoic acids (DHETs) ratio by administration of soluble epoxide hydrolase (sEH) inhibitors in a murine MI model. It has been further hypothesized that these oral administration of such inhibitors are promising to treat cardiovascular disease [45]. One more study [38] has investigated the effect of meloxicam toward plasma and urine lipidome in cats. Cats were administered with meloxicam for 31 days at the dose of 0.3 mg/kg. Plasma and urine lipidome were explored at different time points post-drug administration like 4, 9,13, and 17-days post administration of the drugs. The study depicted increase in the lipids like TG (51:1), TG (49:1), TG (56:6), TG (48:1), TG (54:6) B, SM (d42:2) A from baseline. LPC (16:1), TG (46:4) A, TG (42:3), TG (46:3) were altered in cat urine.

### 3.2. Metabolic Fingerprint in Urine by NSAIDs Administration

While endoscopy is used to determine gastric damage, urine biomarker determination for gastric ulceration is non-invasive as well as cost effective. Three studies investigated metabolic signatures in urine [31,40,41]. In a study by So et al. 2009 [31], pattern recognition was used to understand the detrimental effects of NSAIDs as well as for finding surrogate biomarkers for GI damage induced by NSAIDs. This study had used three different drugs; ibuprofen (800 mg/kg), celecoxib (133 mg/kg), indomethacin (25 mg/kg) and vehicle to unravel the molecular perturbation specific to GI pathophysiology. The drug doses used in the study were 20 times greater than the daily dose commonly used. This study classified the metabolic changes in urine by indomethacin/ibuprofen groups and celecoxib group and aimed that biomarker would proffer screening of GI damage. While allantoin, citrate, 2 oxo-glutamate, taurine, acetate, hippurate and dimethylamine were altered in “ibuprofen/indomethacin” group, the metabolites that are perturbed in “celecoxib” group were citrate, 2 oxo-glutamate, acetate, and hippurate. Allantoin, taurine, and dimethylamine were not changed in “celecoxib” group akin to “indomethacin/ibuprofen” with respect to vehicle group. The differential changes in the metabolite level in indomethacin/ibuprofen vs. celecoxib with respect to vehicle were assumed to be due to changes caused by GI damage although the dose of the drug used was 20 times higher. Interestingly, all three drugs impacted molecules related to energy metabolism like citrate, 2 oxo-glutamate, and acetate.

In 2011, NMR-based untargeted metabolomics was used to understand naproxen-induced toxicity in mice [41]. Male Sprague Dawley rats were used for the metabolomics experiment. Four different doses of naproxen were used, and the damage score was measured. The doses used in the study were 0, 10, 50, and 100 mg/kg naproxen. The damage scores were 0.001 ± 0.003, 0.039 ± 0.007, 0.107 ± 0.022, and 0.163 ± 0.023, respectively. The score was maximum at the dose of 50 and 100 mg/kg of naproxen. The injury was 100 times in naproxen-treated rats as opposed to the vehicle treatment. Citrate, *cis* aconitate, and kynurenate showed a progressive increase in urinary concentration with increase in the dose of the drug. Interestingly, like the previous study, citrate and *cis* aconitate, which are the intermediates of TCA cycle change in concentration due to naproxen administration. Thus, naproxen perturbed similar molecules as indomethacin, ibuprofen, and celecoxib as in the previous study discussed above.

In 2011, metabolomics was further used to understand biomarkers related to indomethacin treatment [40]. In a study by Lv et.al. [40], the experiments were performed in rats. Urine was collected from rats after days 1–3 (day1, day2, day3) post injection of indomethacin. The metabolites were profiled at pre-dose and post-dose administration of indomethacin in rats. Both supervised as well as unsupervised model depicted distinct clustering in the metabolic profile of urine over the time course. There were 20 metabolites that were impacted by indomethacin administration. These metabolites were prostaglandin E2, 2-methylcitric acid, putreanine, (10E,12E)-9-hydroxyoctadeca-10, famotidine, docosanamide, creatinine, pregnenolone, palmitoleic acid, L-carnitine, guanosine, thiamine monophosphate, D-ribulose 5-phosphate, nervonic acid, proline, betaine, spermine, 3 chlorotyrosine, 5-hydroxy-L-tryptophan, 15-keto-prostaglandin F2a and N1-acetylspermidine. The metabolites, 15-keto-prostaglandin F2a and prostaglandin E2 are the COX-derived products. An alteration of these molecules is expected as NSAIDs block the COX enzymes. Additionally, this work also aimed to identify global metabolic perturbations in the tissues; kidney and liver of rats were induced by indomethacin by utilizing ingenuity pathway analysis (IPA). The IPA was used as a tool to phenotype metabolic alterations of the 20 biomarkers confirmed by chemometric analyses. Total of 8 metabolites out of 20 biomarkers qualified for mapping metabolic remodeling induced by indomethacin. This result from IPA demonstrated indomethacin-induced metabolic changes targeting amino acid metabolism and the signaling pathway of the immune response. Carnitine play a role in TCA cycle. It has a role in acyl group translocation in the mitochondria for β oxidation. Similarly, palmitoleic acid provides acetyl CoA for TCA cycle. Thus, alteration of carnitine and palmitoleic acid might suggests dysfunction of mitochondria which has been reported to occur due to NSAIDs administration in several literatures. It is important to note that the time point used in this study was different (24 h, 48 h, 72 h) with respect to previous two studies (5 h and 7 h). This could result in different read out of “metabotype”. However, the change in the fatty acid metabolism is in the line with the previous two literatures.

### 3.3. Metabolic Signatures in Serum/Plasma Induced by NSAIDs

In 2013, Ref. [42] used this discipline to investigate the biomarkers associated with ibuprofen and aspirin in serum in four weeks Sprague Dawley rats. The investigators applied untargeted CE-TOF-MS metabolomics in serum samples. The study involved administration of two different drugs at low dose (3 mg/kg) of aspirin and (8 mg/kg) ibuprofen and high dose (300 mg/kg) of aspirin and (800 mg/kg) ibuprofen followed by analyses after 1 h, 5 h, and 24 h of drug administration. Gastric ulceration was noticed after 5 h of high dose administration of NSAIDs (aspirin/ibuprofen). Metabolites those were perturbed in this case are listed in Table 1. The principal component analysis (PCA) plot demonstrated a maximal difference between control and high dose NSAIDs-administered metabolome at 1 h and 5 h, however, the clustering effect diminished at 24 h post injection. Interestingly, administration of NSAIDs associated with gastric injury in rats is related to an altered level of metabolites like citrate, *cis* aconitate, succinate, acetyl carnitine, 3-hydroxy butanoic acid. Additionally, downregulation of 3-hydroxy butanoic acid, the final product, and marker of fatty acid β-oxidation, indicate that NSAIDs suppressed fatty acid β-oxidation. The data thus suggested a decrease in mitochondrial activity. It is interesting, however, that TCA cycle intermediates like citrate and *cis* aconitate levels were also altered which is similar to previous studies by Ref. [31] and Ref. [41]. Similar to the study by Lv et al. 2011, there was an alteration in fatty acids namely acetyl carnitine and 3- hydroxy butanoic acid. Notably, serum and urine showed a similar alteration in metabolic pathways by NSAIDs which were TCA cycle and fatty acid metabolism.

In 2017, GC-MS-based metabolomics was used to investigate metabolic effects in rats that were fed aspirin (15 mg/kg) or ibuprofen (15 mg/kg) intragastrically for three weeks [44]. A total of 145 features were identified by the investigators in the three groups of rats. Metabolomics was used to understand the toxicities associated with NSAIDs. The most impacted metabolites in serum by both ibuprofen and aspirin were β-hydroxybutyric acid, l-alanine, and aceto-acetic acid. Aceto acetic acid is converted to acetyl CoA by β oxidation. An alteration in the level of this metabolite could suggest a change in mitochondrial activity. This corroborates with the results obtained from the previous studies, which indicated lowered mitochondrial activity during NSAIDs administration. β-hydroxybutyric acid is produced in the liver when glucose metabolism is altered. The metabolic profiles altered by both the drugs are listed in Table 1. One of the interesting conclusions from the study was that the metabolic profiles in host by aspirin and ibuprofen were different. Interestingly, even with different doses of drug being used in this study compared to the previous studies, alteration in fatty acid metabolism remained consistent with regards to previous studies.

### 3.4. Metabolic Signatures in Stomach Induced by NSAIDs

In 2013, Ref. [42] used metabolomics to investigate the biomarkers associated with ibuprofen and aspirin in stomach for four weeks in Sprague Dawley rats. Both low and high doses were used for the study. Proline and hydroxyproline levels in stomach tissues were impacted by high doses of NSAIDs. Interestingly, proline was also perturbed in the study by Lv et al. 2011, albeit in urine and by indomethacin. Furthermore, the study also demonstrated a significant correlation in the metabolites level between the stomach and the serum which suggested that serum biomarkers could be used as a biomarker for stomach injury. In 2019, stomach tissues were used to find metabolic signatures of GI damage [43]. The researchers used NMR to find potential biomarkers due to indomethacin treatment in the stomach tissue. Several metabolites were altered in the stomach. The altered metabolites were pantothenate, isoleucine, spermidine, methionine, acetylcarnitine, trimethylamine, creatinine, carnitine, cis-aconitate, choline, taurine, betaine, glucose, N, N-Dimethylglycine, acetylcholine, tryptophan, and kynurenine. The results obtained from this study were very similar to the studies discussed previously. *Cis* aconitate, the TCA cycle intermediate was also altered as in previous studies such as Ref. [31] and Ref. [41]. Creatinine and betaine levels were also altered as previously reported in [41]. Finally, fatty acid metabolism changes such as carnitine and acetylcarnitine were also altered as reported in previous studies, such as [40,42].

### 3.5. Pathway Analysis

Several groups have used different platforms and drugs to investigate the metabolic perturbations caused by NSAIDs in biofluids and tissues. Interestingly, some of the metabolites are consistently altered in all the studies discussed above. “Pathway analysis” would further be relevant to explore how different pathways are altered in these studies and if there is/are common pathways that are impacted in different studies. The rationale to perform the “pathway analysis” was to get a robust change in specific pathway (s) if any, across all the studies. This is thus performed on the data of the published literatures that are discussed above.

**Figure 1:**
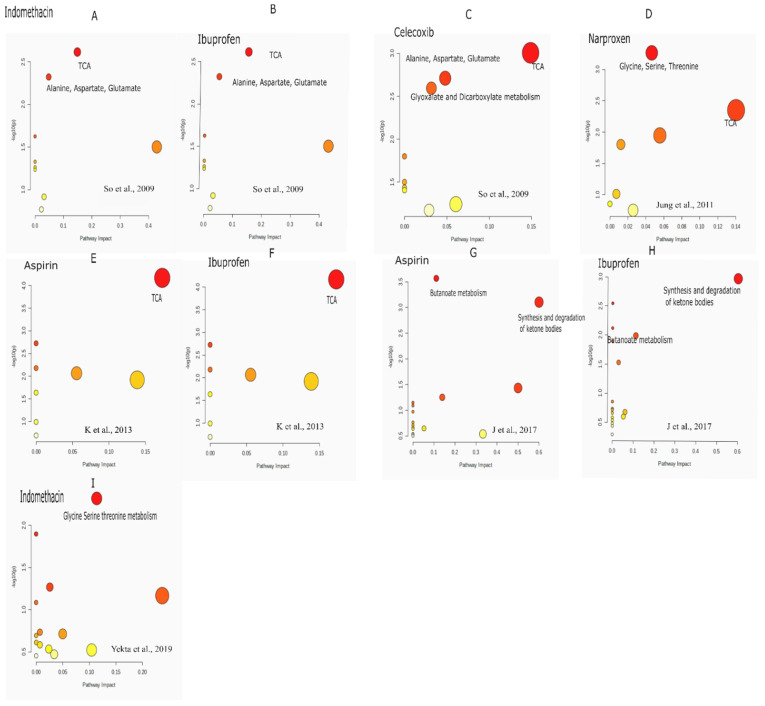
Pathway analysis performed on the published literature. (**A**–**C**) are pathway analysis done from the literature, So et al.,2009; (**D**) Jung et al.,2011; (**E**,**F**) K et al. 2013; (**G**,**H**) J et al. 2017. (**I**) Yekta et al. 2019.

**Figure 2:**
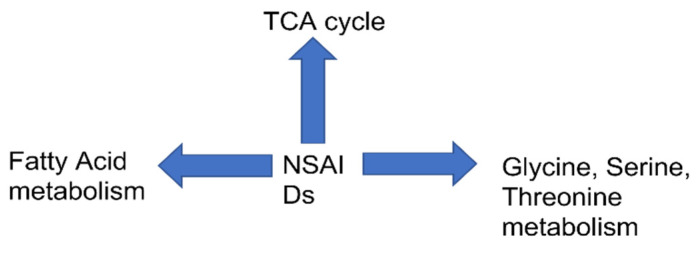
Global metabolic perturbation in host by NSAIDs.

The “pathway analysis” is performed using metabolomics data from the literature by So et al. 2009. TCA cycle is the most significant pathway impacted by all the drugs: indomethacin/ibuprofen and celecoxib with FDR 0.19 and 0.07 respectively as seen in Figure 1. Additionally, “alanine, aspartate, glutamate” pathway was altered by celecoxib with FDR of 0.07 and by indomethacin/ibuprofen with FDR of 0.19 (Figure 1). Glyoxylate and dicarboxylate metabolism with FDR of 0.07 was altered in celecoxib group. “Pathway analysis” performed on the data by Jung et.al., 2011 shows that the maximally impacted pathways were “TCA cycle” with FDR of 0.18 and “glycine serine and threonine metabolism” with FDR of 0.04 as shown in Figure 1. The “pathway analysis” was not significant for the data published by “Lv et.al., 2011” so it is not discussed here. Further, the data from the paper by K et al. 2013 is used for “pathway analysis”. As seen in Figure 1, TCA cycle was impacted maximally after 1–5 h post administration of aspirin and ibuprofen. The FDR for TCA cycle is 0.0056 for both the drugs. In 2017, GC-MS based metabolomics was used to investigate metabolic effects in rats that were fed aspirin (15 mg/kg) or ibuprofen (15 mg/kg) intragastrically for three weeks [44]. The significant pathways are “synthesis and degradation of ketone bodies” with FDR 0.08 in ibuprofen and FDR of 0.03 in aspirin group respectively. “Butanoate metabolism” was impacted due to administration of both the drugs in this study with FDR of 0.02 in “aspirin group” and 0.21 in “ibuprofen group”. Additionally, the investigators also found *cis* aconitate alterations which could be due to TCA cycle perturbations as *cis* aconitate is an intermediate of TCA cycle. Further, the data from Yekta et.al, was used for pathway analysis. The analysis showed “glycine serine threonine” metabolism to be impacted mostly by Indomethacin. Figure 2 summarizes the pathway level alterations by NSAIDs.

## 4. Discussion and Future Research

Several groups have investigated metabolic fingerprinting due to NSAIDs administration in rats. Some of the studies have used urine and serum as the biofluid of interest while two have used stomach tissues to get into mechanistic details of the GI-related injury in NSAIDs administration. Another motivation for investigating host metabolism in NSAIDs was to discover biomarker candidates for monitoring NSAIDs administration.

Some of the studies documented the effects of drugs at very high doses like ibuprofen (800 mg/kg) and indomethacin (25 mg/kg) in rats. These doses were greater than the dose required to induce GI pathology (20 times higher). Lanza evaluated the effect of low (500 mg/kg) and high doses (750 mg/kg) of naproxen for 7 days on gut injury [46]. Human gastric damage invoked by low dose and high dose was similar to 10 mg/kg and 100 mg/kg as in rats. Ibuprofen is also related to ulcers formation in gut of monkeys. Monkeys given 100 mg/kg intravenously per 24 h had stomach and duodenal ulcers [47].

The metabolomics studies that explored the molecular fingerprints of GI lesions induced by NSAIDs specifically show that naproxen could thus be theoretically translated to humans. Thus, biomarkers of GI lesions at the above dose could be equivalent in humans. However, no study has validated this finding so far. The limitations of all the studies discussed in this paper are thus, that the findings are not yet validated in humans. Furthermore, rofecoxibadministration in mice reduced bleeding time which is associated with pathogenesis of myocardial infarction (MI). This was associated with biomarker of 20-HETE. In humans, the bleeding time in myocardial infarction is reported to be shorter compared to the ones without MI, however, no metabolomics experiment has been conducted in humans to validate the biomarker findings in mice in this front.

The studies investigating alteration in lipidomics by NSAIDs clearly suggested a perturbation in lipid profile in NSAIDs user. NSAIDs are known to cause stress in endoplasmic reticulum, the major site for lipid synthesis and degradation [48,49]. The change in the lipid profile could be driven by the alternation in endoplasmic reticulum, however, more involved experiments are needed to address this issue.

We have performed the “pathway analysis” to get an understanding of robust change of metabolism in host due to NSAIDs administration. In three independent studies as discussed above, it is evident that TCA cycle was the most impactful pathway to be altered by NSAIDs. The pathway impact ranged between 0.1 and 0.2, with FDR cutoff between 0.001 and 0.2. These studies involved different drugs, indomethacin, ibuprofen and celecoxib, naproxen, and aspirin. TCA cycle is impacted by different types of NSAIDs; namely non-specific NSAIDs, COX-1, and COX-2-specific NSAIDs. This could be related to mitochondrial dysfunction as TCA cycle primarily takes place in mitochondria. From our analysis, we found TCA cycle to be impacted in most studies, irrespective of the fact that the studies were conducted by different research groups, different drugs, and doses. The table clearly shows that the TCA cycle intermediates (e.g., citrate, *cis* aconitate, succinate etc.,) are impacted maximally in all the studies. Thus, the mitochondrial dysfunction could be one of the critical factors for such perturbations in the metabolic pathways. NSAIDs have been reported to influence the uncoupling of oxidative phosphorylation, increase in resting state respiration, and disruption of mitochondrial transmembrane potential [50,51]. These alteration in mitochondrial energy production by NSAIDs could induce tissue damage. Additionally, “glycine serine and threonine” metabolism are impacted in two independent studies. One of the possible reasons could be the remodeling of glucose metabolism for the synthesis of serine in NSAIDs. As we discussed before, TCA is a potential target pathway in NSIADs, which causes a change in acetyl CoA, the initial compound of TCA. This might in turn cause a shift in the metabolism of glucose, shifting it more toward production of serine.

Additionally, we found change in fatty acid metabolism. Several studies mentioned above show fatty acid intermediates to be impacted. An excess of acetyl CoA, which may stem from TCA cycle dysfunction can lead to alteration in “synthesis and degradation of ketone bodies.” Other than this, butanoate metabolism was also impacted by NSAIDs administration. Butyrate is produced in the colon by the fermentation of carbohydrates by bacteria. Previous studies on NSAIDs have shown to alter butyrate as being associated with decrease in markers of inflammation [52]. Further, dysbiosis is known to be caused by NSAIDs [53] which might affect “butyrate metabolism.”

Other than this, “alanine, aspartate, and glutamate” metabolism is also impacted by NSAIDs administration in rodents. This along with “glycine serine and threonine pathway could be implicated in TCA cycle perturbations. Alanine, glycine, serine could feed into TCA cycle by the formation of pyruvate which subsequently forms acetyl CoA, the initial component of TCA cycle. Aspartate can also feed into TCA cycle via the formation of oxaloacetate. Overall, the metabolic pathway analysis could mean a change in TCA cycle, amino acid metabolism, and fatty acid metabolism.

The future studies should be thus directed toward understanding the perturbations in the TCA cycle, amino acid, as well as fatty acid metabolism by NSAIDs administration. Importantly, the role of endoplasmic reticulum and mitochondria in alterations of the pathway requires further study.

Further studies can explore the mechanism by which TCA is dysregulated by NSAIDs and possible involvement of other organs such as gut, liver, heart, and kidney to shed light on the molecular defects in the pathogenesis of GI. To understand the therapeutic targets of the TCA cycle intermediates, interventions at the tissue level are warranted as urine analysis does not allow predicting the actual target organ where one should use the drug. This further requires many tissues to be harvested from mice administered with NSAIDs. Furthermore, diverse analytical platforms need to be involved to cancel bias, to understand the mechanistic aspects involved in the TCA cycle. A further insight into the specific enzymes in the pathway will delineate the exact nature of perturbations in the NSAIDs administration and shed light on the mechanistic aspect.

## Figures and Tables

**Table 1:** List of the metabolites that are altered due to NSAIDs administration in rats as obtained from metabolomics studies. (up and dn denote upregulated and downregulated respectively, nm denote not mentioned in manuscript.).

Drugs	Biofluids/ Tissues	Dose	Timepoint	Species	Metabolites	Analytical Platform	Publications
Celecoxib	Urine	133 mg/kg	5 h	Rats	Citrate(dn), 2-oxoglutarate(dn), Acetate(dn), Hippurate(dn)	NMR	[31]
Indomethacin	Urine	25 mg/kg	5 h	Rats	Allantoin(up), Citrate(dn), 2-Oxoglutarate(dn), Taurine(dn), Hippurate(dn), Dimethylamine(up)	NMR	[31]
Ibuprofen	Urine	800 mg/kg	5 h	Rats	Allantoin(up), Citrate(dn), 2-Oxoglutarate(dn), Taurine(dn), Hippurate(dn), Dimethylamine(up)	NMR	[31]
Naproxen	Urine	10 mg/kg-100 mg/kg	7 h	Rats	Kynurenate (up), pantothenate(dn), citrate (up), creatine(up), creatine(up) phosphate (up), cis-aconitate (up), choline (dn), and betaine(up)	NMR	[41]
Indomethacin	Urine	25 mg/kg	0–24 h, 24–48 h, 48–72 h	Rats	Prostaglandin E2(dn), 2-methylcitric acid(dn), putreanine(up), (10E,12E)-9-hydroxyoctadeca-10, famotidine(dn), docosanamide(up), creatinine(up), pregnenolone(dn), palmitoleic acid(up), l-carnitine(dn), guanosine(dn), thiamine monophosphate(dn), d-ribulose 5-phosphate(up), nervonic acid(up), proline betaine(dn), spermine(up), 3 chlorotyrosine(up), 5-hydroxy-l-tryptophan(up), 15-keto-prostaglandin F2a(dn) and N1-acetylspermidine(dn).	LC/MS	[40]
Aspirin	Stomach	300 mg/kg	1 h	Rats	Citrate(dn), *O*-Acetylcarnitine(dn), 3hydroxybutanoic acid(dn), Proline(dn), Hydroxyproline(dn)	CE-TOF-MS	[42]
Aspirin	Stomach	300 mg/kg	5 h	Rats	Citrate(dn), cis-Aconitate(dn), succinate(dn) O-Acetylcarnitine(dn), 3hydroxybutanoic acid(dn), Hydroxyproline(dn)	CE-TOF-MS	[42]
Ibuprofen	Stomach	800 mg/kg	1 h	Rats	Citrate(dn), succinate(dn) O-Acetylcarnitine(dn), 3hydroxybutanoic acid(dn), Hydroxyproline(dn)	CE-TOF-MS	[42]
Ibuprofen	Stomach	800 mg/kg	5 h	Rats	Citrate(dn), cis-Aconitate(dn), succinate(dn) O-Acetylcarnitine(dn), proline(dn), Hydroxyproline(dn)	CE-TOF-MS	[42]
Aspirin	Serum	300 mg/kg	1 h	Rats	3-Hydroxy butanoic acid(dn), proline(dn), hydroxyproline(dn)	CE-TOF-MS	[42]
Aspirin	Serum	300 mg/kg	5 h	Rats	Cis-Aconitate(dn), o acetyl carnitine(dn), 3-Hydroxy butanoic acid(dn), proline(dn), hydroxyproline(dn)	CE-TOF-MS	[42]
Ibuprofen	Serum	800 mg/kg	1 h	Rats	3-Hydroxy butanoic acid(dn),	CE-TOF-MS	[42]
Ibuprofen	Serum	800 mg/kg	5 h	Rats	Succinate(dn), o-acetyl carnitine(dn), 3-Hydroxy butanoic acid(dn), proline(dn), hydroxyproline(dn)	CE-TOF-MS	[42]
Ibuprofen	Serum	15 mg/kg	1–3 week	Rats	Acetoacetic acid(nm), l-alanine(nm), Trihydroxybutyric acid(nm), Galacturonic acid(nm), Propanedioic acid(nm), Acetic acid(nm), Ethanedioic acid(nm), Galacturonic acid(nm), L-valine(nm), Mannonic acid(nm), Urea(nm), D-galactose(nm), Ethanedioic acid(nm), l-isoleucine(nm), Propanoic acid(nm), Butenoic acid(nm), d-glucose(nm), L-norvaline(nm), Hexanoic acid(nm), Acetamide(nm).	GC-MS	[44]
Aspirin	Serum	15 mg/kg	1–3 week	Rats	Trihydroxybutyric acid(nm), l-alanine(nm), Galacturonic acid(nm), D-galactose(nm), l-alanine(nm), Acetamide(nm), Propanedioic acid Acetoacetic acid(nm), Butanoic acid(nm), Arachidonic acid(nm), Ethanedioic acid(nm), L-tyrosine(nm), d-glucose(nm), Propanoic acid(nm), Hexanoic acid(nm).	GC-MS	[44]
Indomethacin	stomach	45 mg/kg	6 h	Rats	Choline(dn), Cis-aconitate(dn), Tryptophan(dn), Spermidine(dn), Trimethylamine(up), N,N-Dimethylglycine(dn), Acetylcarnitine(dn) Creatinine(dn), Pantothenate(dn), Betaine(dn), Carnitine(dn), Isoleucine(dn), Glucose(dn), Kynurenine(dn), Methionine(dn), Acetylcholine(up)	NMR	[43]

## Data Availability

Not applicable.
